# Oral dysfunction as a cause of malocclusion

**DOI:** 10.1111/ocr.12277

**Published:** 2019-05-10

**Authors:** Linda D'Onofrio

**Affiliations:** ^1^ Oregon Health and Sciences University School of Dentistry Portland Oregon

**Keywords:** breastfeeding, malocclusion, oral dysfunction, orofacial myofunctional disorder

## Abstract

This narrative review surveys current research demonstrating how oral dysfunction can escalate into malocclusion, acquired craniofacial disorder and contribute to generational dysfunction, disorder and disease.

**Introduction:**

Baseline orthodontic consultations are generally recommended beginning age seven. However, the dysmorphic changes that result in malocclusion are often evident years earlier. Similarly, following orthodontic treatment, patients require permanent retention when the bite is not stable, and without such retention, the malocclusion can return.

**Setting and Population:**

Narrative review article including research on infants, children and adults.

**Materials and Methods:**

This review is a brief survey of the symptomology of orofacial myofunctional disorder and outlines 10 areas of oral function that impact occlusal and facial development: breastfeeding, airway obstruction, soft tissue restriction, mouth breathing, oral resting posture, oral habits, swallowing, chewing, the impact of orofacial myofunctional disorder (OMD) over time and maternal oral dysfunction on the developing foetus.

**Conclusion:**

Malocclusions and their acquired craniofacial dysmorphology are the result of chronic oral dysfunction and OMD. In order to achieve long‐term stability of the face, it is critical to understand the underlying pathologies contributing to malocclusion, open bite and hard palate collapse.

## 1 INTRODUCTION

Most infants are beautiful because most children are born with normal craniofacial shape, normal jaw relationship and potential for optimal airway. In most newborn faces, the alveolar process easily accommodates the tongue and all future teeth.

Nevertheless, orthodontists see multitudes of children with abnormal jaw relationship, steep mandibular angle (SMA), anterior open bite (AOB), high narrow palate (HNP), posterior cross bite (PCB) and suboptimal facial development. While orthodontic referrals may begin at age seven, the facial dysmorphology is often evident years earlier. When oral dysfunction goes untreated, orofacial myofunctional disorder (OMD) can result.

Orofacial myofunctional disorder includes dysfunction of the lips, jaw, tongue and/or oropharynx that interferes with normal growth, development or function of other oral structures, the consequence of a sequence of events or lack of intervention at critical periods, that result in malocclusion and suboptimal facial development.

Oral dysfunction can begin with our very first breath and with our very first feeding.^1^ OMD can become apparent as children learn to speak^2^ and transition to table food.^3^ Most children with OMD are diagnosed after experiencing articulation disorder, sleep‐disordered breathing (SDB)^4^ or malocclusion.^5^ Orthodontic relapse, obstructive sleep apnoea (OSA) and temporomandibular disorder^6^ are predictable consequences of long‐term oral dysfunction and OMD.

This manuscript provides a brief narrative survey of ten areas of oral function related to occlusal and facial development: breastfeeding, airway obstruction, soft tissue restriction, mouth breathing, oral resting posture, oral habits, swallowing, chewing, OMD over time and maternal oral dysfunction on the developing foetus.

## 2 IMPACT OF BREASTFEEDING

Breastfeeding is the first and perhaps most critical experience to facial development. Unlike with bottle feeding, infants draw the breast deep into the mouth and the breast expands and shapes the hard palate through repeated pressure and peristaltic wave.^7^ Breastfeeding requires jaw compression, which helps develop better masseter muscles than does bottle feeding.^8^

Children exclusively breastfed appeared to have a lower incidence of malocclusion later in life when compared to bottle‐fed babies. Studies demonstrated exclusive breastfeeding had an inverse correlation to AOB,^9^ PCB, overjet and other malocclusions.^10^, ^11^ And the longer children nursed, the better. Children who breastfed over 6 months had lower chance of overjet, and they demonstrated wider inter‐canine and inter‐molar width.^12^ A number of studies also found that extended breastfeeding continued to decrease the risk of malocclusion, and the longer a child breastfed, the less likely they were to have malocclusion.^13^, ^14^

## 3 IMPACT OF AIRWAY OBSTRUCTION

A very young infant will typically breathe quietly with lips closed. But even in early infancy, there are a number of factors that can interrupt this process and change the course of craniofacial growth. Airway obstruction has many aetiologies and is not uncommon in early childhood.

Allergic rhinitis, with and without oral habits, has been implicated in both anterior and posterior open bites.^15^ The condition commonly known as ‘long adenoid face’ is marked by enlarged tonsils or adenoids that accompany a retrognathic jaw, SMA and with larger lower anterior face height.^16^ Otitis media is correlated with HNP and PCB.^17^ Septal deviation can result in a HNP, demonstrating the inter‐relationship of these facial features.^18^

## 4 IMPACT OF SOFT TISSUE RESTRICTION

Research on ankyloglossia diagnosis and its impact on oral function is growing because of its implication in OSA.^4^ The frenulum's upper and lower insertion points, its thickness and flexibility and length of free anterior tongue all impact lingual range of motion and oral function. Ankyloglossia has been correlated with reduced inter‐canine and inter‐molar width, SMA, HNP, AOB, overjet and incisor spacing.^19^ The vertical lift to the hard palate, and not horizontal extension past the incisors, is the most accurate measure of normal lingual function.^20^

The existence of labial frenula is not a symptom of dysmorphology, but variations in the insertion points, thickness and its contribution to trapping liquid and food can have a negative impact on oral and dental development. Maxillary labial ties can make breastfeeding difficult and painful for the mother when the upper lip does not create enough of a flange to adequately draw in more of the nipple.^21^ As teeth develop, central incisors may separate, rotate or flare in response to a low fibrous frenulum.^22^ Buccal ties are the least researched of the oral frenula and their impact on gingival recession and maxillary growth are not well documented.^23^ Pronounced buccal frenula contribute to pocketing of food in the vestibules.

## 5 IMPACT OF MOUTH BREATHING

If the impact of airway obstruction, soft tissue enlargement and/or soft tissue restriction is great enough, nasal breathing may not be adequate for muscular and cognitive functions, and a pattern of mouth breathing can develop. The sinuses experience their largest growth in early childhood, and nasal breathing activates growth in occipital and nasal joints and sutures of the facial bones.^24^ Mouth breathing encourages a lower jaw posture which can change directional growth over time.

When compared to those with nasal breathing patterns, mouth breathing was more highly correlated to HNP, PCB and AOB.^25^ Mouth breathing during the critical facial growth period was associated with a ‘clockwise’ rotation of the mandible and an increase in lower anterior face height.^5^

Mouth breathing not only changes the anterior of the face, but also changes the shape of the oropharyngeal airway. With an increase in anterior face height, there is often a decrease in posterior height. Mouth breathing has been associated with smaller retropalatal and retroglossal areas, and lengthening of the pharynx, a risk factor for OSA.^26^

Orofacial myofunctional disorder should be viewed on a continuum, and as oral dysfunction influences the growth of oral structures, the structure impacts oral function in return. Mouth breathing at night contributes to other symptoms of SDB, including snoring. Children who snore are more likely to have HNP and PCB.^27^ Mouth breathing at night, without any other symptoms, ‘is a risk factor for OSA, and is associated with increased disease severity and upper airway collapsibility.’^28^

Once a child has been diagnosed with OSA, they often present with extreme malocclusions and dysmorphology. ‘Children with diagnosed OSA had a significantly increased overjet, a reduced overbite, narrower upper and shorter lower dental arches when compared with the controls. Snoring children had similar but not as significant differences as OSA children when compared to controls. There were more children with an AOB in the OSA group and with a Class II or asymmetric molar relationship in the groups of OSA and snoring subjects compared with non‐obstructed controls.’^29^ This study asserts that structural changes are caused by long‐term functional changes in the head, neck and tongue in order to maintain a patent airway during sleep.^29^

Breastfeeding reduces the chances of a child developing SDB. A study of school‐aged children found those who were breastfed for only a few months had less incidence of snoring and OSA than those who were bottle‐fed.^30^

## 6 IMPACT OF ORAL RESTING POSTURES

‘The ability to breathe effortlessly and quietly through the nose with the tongue suctioned up and the lips gently closed is essential to optimal craniofacial growth and development.’^31^ Muscular pressure on facial bones, or the lack thereof, can influence directional growth over time. Open lip posture can encourage upper incisor flaring.^32^ Lingual‐palatal stability maintains the palatal arch and supports the mid and lower anterior face. Low lingual resting posture has been correlated with both Class II and Class III malocclusions.^33^ The static lingual posture at rest slowly changes the face, the swallowing pattern and occlusion.

## 7 IMPACT OF ORAL HABITS

Most dentists and orthodontists understand that sucking and chewing habits contribute to AOB and PCB.^15^, ^34^ However, instead of treating thumb sucking as an aberrant habit, evidence indicates this behaviour may be a symptom of airway obstruction^15^ and/or ankyloglossia.^35^

In addition to inappropriate and uneven pressure into the hard palate and alveolar process, oral habits contribute to keeping the tongue low and forward in the mouth, which promotes an open mouth resting posture and the cascade of effects that can follow.^35^ As expected, the longer oral habits continue, the more severe the malocclusion.^36^

The occurrence of oral habits may be in response to an appropriate biological need and breastfeeding may be the best prevention. An inverse relationship was found between the duration of breastfeeding and the occurrence of oral habits.^37^ Palatal stimulation appears to be a necessary part of facial and cognitive development, and by allowing infants to experience it through breastfeeding may not only promote better facial development, but also reduce the likelihood of maladaptive oral habits.^38^

## 8 IMPACT OF SWALLOWING

Atypical swallowing develops as a compensatory movement pattern when normal movement is inhibited in some way. A tongue thrust swallow involves excessive perioral effort and the tongue exerts forward and/or lateral pressure into the teeth, rather than vertical pressure into the hard palate with a front to back motion.^39^ Lingual‐palatal stabilization for the swallow is far weaker in children with PCB.^40^ This swallowing pattern reinforces a low resting posture, contributing to HNP, PCB and further malocclusion.^41^

## 9 IMPACT OF CHEWING

Chewing begins in the first year of life and provides early sensory‐motor awareness, oral proprioception and a foundation for normal oral movement needed for speech. In addition to aiding in the digestion of food, chewing stabilizes the temporomandibular joint^42^ and regulates bone growth.^43^ Chewing helps reduce psychological stress,^44^ improve attention^45^ and increase cognition.^46^

Non‐nutritive chewing of objects, imbalanced chewing and inefficient chewing can contribute to the development of malocclusion. Researchers are now speculating on how our diet has evolved over the centuries and its possible contribution to craniofacial changes, specifically to retrognathia.^47^ Softer foods require less chewing and less bite force. Masseter orientation angle and bite force were found to be correlated to different malocclusions, with Class III showing to have the greatest bite force.^48^ Children with PCB demonstrated reduced bite force and unbalanced jaw function.^49^

Because one symptom of OMD can contribute to the development of another, it is logical to assume that mouth breathing and oral habits can negatively impact masseter development. Compared to nasal breathing, mouth breathing is shown to reduce the chewing stroke count and chewing cycles.^50^ Children with oral habits produced less bite force than children without such habits.^51^

## 10 IMPACT OF OMD OVER TIME

As stated above, OMD is often the result of a sequence of events or lack of intervention at critical periods. The impact is cumulative. Children with low rates of breastfeeding, with oral habits and mouth breathing during sleep present with more malocclusions.^52^ When OMD occurs during childhood, this disorder then becomes a contributing factor in other diseases and disorders.^28^, ^53^, ^54^, ^55^

The long‐term concerns go well beyond poor facial aesthetics. Unresolved OMD can contribute to serious dental and medical conditions that threaten the quality and length of a person's life. If the jaw is rotated back into the airway and the hard palate invades and deviates the sinuses,^56^ it may be difficult to breathe nasally. As the face grows, unbalanced pressure on the craniofacial bones can contribute to temporomandibular dysfunction. Airway‐based malocclusions and those correlated to SDB are further complicated by co‐occurring symptoms including clenching and grinding, regarded together as bruxing. Bruxing can contribute to facial pain and tooth damage.

New research into SDB and OSA links poor sleep and airway obstruction to daytime behavioural disorders in children.^53^ The relationship between SDB and increased risk for academic and social failure is also well documented.^54^ By the time a child reaches their teens, their dysmorphic facial structure may put them at permanent risk for a lifetime of airway function disorders.^31^ A study of facial measurements of over 4000 teens concluded, ‘the combination of a long face, reduced nose prominence and width, and a retrognathic mandible may be diagnostic facial features of SDB that may warrant a referral to specialists for the evaluation of other clinical symptoms of SDB.^55^

## 11 IMPACT OF MATERNAL ORAL DYSFUNCTION ON THE DEVELOPING FOETUS

Sleep‐disordered breathing is common during pregnancy and is linked to hypertension, gestational diabetes, pre‐eclampsia and foetal growth retardation.^57^ A retrospective study of over 300,000 women at one military treatment facility found that women diagnosed with OSA had higher rates of cesarean delivery, gestational hypertension, pre‐eclampsia and preterm delivery,^58^ and there were indications that maternal OSA was a direct cause of foetal distress.^59^

Research suggests infants of mothers with OSA are more likely to be born with a retrognathic jaw and HNP and premature infants appear to be more prone to PCB.^60^ Those born small for gestational age were noted to have a short anterior cranial base, increased lower anterior face height, small retrognathic jaws with SMA and small maxilla. Interestingly, this same study found dental age was not delayed, which extrapolates to normal sized teeth in an underdeveloped face.^61^ Instead of starting out with beautifully aligned facial morphology, these infants begin life at risk for OMD and all the consequences that follow.

## 12 CONCLUSION: PREVENTION OF MALOCCLUSION

Orthodontists must advance their thinking in how, when and why they are treating their patients. Closing an open bite without resolving its underlying pathology increases the risk of orthodontic relapse.^62^ The signs and symptoms of OMD can appear in the first weeks of life but can also occur at any point in the lifespan. In addition to providing structural solutions to problems once they occur, dentists and orthodontists must play a proactive role in preventing acquired craniofacial disorders and supporting optimal craniofacial growth.

In response to a growing body of scientific and clinical evidence, all medical and dental professionals have a responsibility to screen for daytime and nocturnal breathing disorders, for enlarged and restricted oral tissue in patients of all ages, and for feeding and oral dysfunction early in life. Beautiful babies were meant to grow up to be beautiful adults.
